# Prevalence of Vitamin D Deficiency and Its Associated Work-Related Factors among Indoor Workers in a Multi-Ethnic Southeast Asian Country

**DOI:** 10.3390/ijerph17010164

**Published:** 2019-12-25

**Authors:** Ushashree Divakar, Thirunavukkarasu Sathish, Michael Soljak, Ram Bajpai, Gerard Dunleavy, Nanthini Visvalingam, Nuraini Nazeha, Chee Kiong Soh, Georgios Christopoulos, Josip Car

**Affiliations:** 1 Centre for Population Health Sciences, Lee Kong Chian School of Medicine, Nanyang Technological University, 11 Mandalay Road, Singapore 308232, Singapore; ushashreedivakar@gmail.com (U.D.); m.soljak@imperial.ac.uk (M.S.); r.bajpai@keele.ac.uk (R.B.); gerard.dunleavy@ntu.edu.sg (G.D.); nv@rainmaking.io (N.V.); nuraini.nazeha@ntu.edu.sg (N.N.); josip.car@ntu.edu.sg (J.C.); 2 Population Health Research Institute, McMaster University, 237 Barton Street East, Hamilton, ON L8L 2X2, Canada; 3 Department of Primary Care & Public Health, School of Public Health, Imperial College, London SW7 2AZ, UK; 4 Research institute for Primary Care and Health Sciences, Keele University, Staffordshire ST5 5BG, UK; 5 School of Civil and Environmental Engineering, College of Engineering, Nanyang Technological University Singapore, 50 Nanyang Avenue, Singapore 639798, Singapore; CSOHCK@ntu.edu.sg; 6 Decision, Environmental and Organizational Neuroscience Lab, Culture Science Institute, Nanyang Business School, Nanyang Technological University, Singapore 639798, Singapore; CGeorgios@ntu.edu.sg; 7 Division of Leadership, Management and Organization, Nanyang Business School, College of Business, Nanyang Technological University, Singapore 639798, Singapore

**Keywords:** vitamin D deficiency, indoor workers, cross-sectional study, workplace, Singapore

## Abstract

Little is known about the effect of working conditions on vitamin D status in Southeast Asia, where vitamin D deficiency is common despite the presence of sunlight all year round in most places. We examined the prevalence of vitamin D deficiency and its associated work-related factors among indoor workers using the data of 213 participants (aged ≥21 years) from a workplace cohort study in Singapore. Vitamin D deficiency was defined as serum 25-hydroxyvitamin D (25(OH)D) concentration <50 nmol/L. Data on work-related factors, socio-demographic characteristics, and lifestyle habits were collected using standardized questionnaires. Clinical and biochemical measurements were performed using standard tools and protocols. Multivariate Poisson regression was used to examine the independent association of work-related factors with vitamin D deficiency. Mean serum 25(OH)D concentration was 59.6 nmol/L. The prevalence of vitamin D deficiency was 32.9% (95% confidence interval (CI): 26.6–39.6%). In the multivariate analysis, office workers (prevalence ratio (PR): 2.16, 95% CI: 1.12–4.16 versus control room workers), workshop workers (PR: 2.25, 95% CI: 1.05–4.81 versus control room workers), and night shift workers (PR: 1.31, 95% CI: 1.03–1.67) were at a greater risk for vitamin D deficiency. Workplace policies and wellness programs should encourage workers to take regular breaks to go outdoors for sunlight exposure and to consume adequate amounts of vitamin D-rich foods to maintain optimal vitamin D levels.

## 1. Introduction

Vitamin D, also known as the “sunshine” hormone, is responsible for calcium homeostasis, cell proliferation and differentiation, and regulation of the immune and the neuromuscular systems [1]. Vitamin D deficiency or insufficiency is a major health problem affecting an estimated one billion individuals globally [2]. Vitamin D deficiency has been associated with a range of health outcomes, including impaired bone homeostasis, autoimmune diseases, infectious diseases, dementia, Alzheimer’s disease, cancers, diabetes, cardiovascular disease, inflammatory bowel disease, and all-cause mortality [3]. However, many of these associations have subsequently been questioned [4]. A recent meta-analysis of 52 trials of vitamin D supplementation with a total of 75,454 participants found that supplementation was not associated with all cause deaths (risk ratio 0.98, 95% confidence interval 0.95 to 1.02), cardiovascular deaths (0.98, 0.88 to 1.08), or non-cancer, non-cardiovascular deaths (1.05, 0.93 to 1.18), although it did significantly reduce the risk of cancer death by 16% (0.84, 0.74 to 0.95) [5]. This is at least the 15th meta-analysis of vitamin D supplements and mortality or a subgroup of mortality in the past 12 years, and there remains no consistent evidence that vitamin D has any meaningful clinical benefit for these outcomes.

Much of vitamin D (90%) is synthesized in the skin following exposure to ultraviolet B (UV-B) radiation from sunlight, while the rest (10%) is obtained from foods such as oily fish, egg yolks, fortified milk and juices, and dietary supplements [6]. Vitamin D obtained from these sources is converted in the liver to 25-hydroxyvitamin D (25(OH)D), which is the major circulating metabolite and the best available indicator for vitamin D status [7]. As the endogenous production of vitamin D is highly dependent on sunlight, factors associated with spending less time outdoors could adversely influence vitamin D levels.

A recent systematic review of 71 studies from across the globe showed that occupation is a major factor contributing to sub-optimal vitamin D levels, and indoor workers are at a greater risk compared to outdoor workers; more than three-fourths (78%) of indoor workers were found to be vitamin D deficient compared to less than half (48%) of outdoor workers [8]. Indoor workers spend a significant amount of time working indoors without sunshine exposure. Furthermore, due to the conventional working time of indoor workers, they are exposed to sunlight mostly during mornings and evenings, when the intensity of UV-B exposure is relatively low. Sunlight deprivation among indoor workers puts them at high risk of developing vitamin D deficiency and its associated health risks.

In Southeast Asia, despite the presence of sunlight all year round in most places, several studies have reported a high prevalence of vitamin D deficiency [9]. Sun-protection behaviors such as wearing hats, sunscreen use, staying in the shade, wearing long sleeves or using an umbrella are common in this region. While the association between vitamin D deficiency and socio-demographic, lifestyle, clinical, and biochemical characteristics among the general population in Southeast Asia has been previously reported [10,11,12,13,14,15], relatively little is known about the effect of working conditions on vitamin D status. Understanding the work-related factors influencing vitamin D status can inform workplace policy and strategies to improve vitamin D levels of workers, and ensure improved health outcomes.

Singapore is a multi-ethnic Southeast Asian country with Chinese being the majority followed by Malays, Indians, and other Asian ethnicities. Singapore (1°18′N) lies close to the equator and has a tropical climate with significant amounts of rainfall, high and stable temperatures, and high humidity throughout the year [16]. Although there have been studies on vitamin D deficiency in Singapore among the general population [17], patients with hip fracture [10], and elderly inpatients [18], to our knowledge, there are no published data in the working population of Singapore. Therefore, we aimed to examine the prevalence of vitamin D deficiency and its associated work-related factors among indoor workers in Singapore.

## 2. Materials and Methods

### 2.1. Study Design and Sample

Between August 2016 and January 2017, a cohort was established by recruiting participants aged 21 years and above from four workplaces in Singapore. The main objective of the study was to examine the effects of working in underground spaces on sleep quality and melatonin levels. Details of the cohort study design are published elsewhere [19]. Briefly, two railway companies, a cooling plant, and a university were recruited. Employees in these workplaces were invited to participate in the study via meetings, worksite posters, and emails. Those willing to participate were screened for eligibility using the following criteria: aged 21 years and above, should speak and read English, should be working for at least four hours per day, not pregnant, had not travelled overseas across a different time zone on a monthly basis over the previous six months, and not taking sleep medications, exogenous melatonin, or steroids. A total of 516 employees were screened, and 464 of whom were found to be eligible and enrolled in the study. Participants were followed-up after three and 12 months from baseline. Of 464 participants at baseline, 334 (72%) participated in the 12-month follow-up, and 213 (64%) provided blood samples for 25(OH)D, who were included in this analysis. Data collection for this paper was conducted between August 2017 and March 2018. In Singapore, rainy seasons are from December to early March and from June to September, and summer is during the inter-monsoon periods [16]. Ultraviolet Index in Singapore during the data collection period was ~7.5 (high) [20].

### 2.2. Measurements

#### 2.2.1. Serum D 25(OH)D Levels

Venous blood samples were collected from participants in a fasting state (at least eight hours) by trained phlebotomists. Blood samples were centrifuged and transported in boxes with dry ice to an internationally accredited laboratory for analysis. Serum 25(OH)D is the primary circulating form of vitamin D with a half-life of approximately two to three weeks and is the best indicator of the body’s vitamin D status [6]. Serum 25(OH)D concentrations were measured using the chemiluminescence immunoassay method on a Cobas E 411 analyzer with kits supplied by Roche Diagnostics, Switzerland. Although there is no consensus on the definition of vitamin D deficiency, serum 25(OH)D) levels <50 nmol/L is widely used in studies, and recommended by the Endocrine Society clinical practice guideline [6] and the US Institute of Medicine [1].

#### 2.2.2. Work-Related Factors

Self-administered questionnaires were used to collect data on number of years employed in the current company, work location (underground or aboveground), job type (control room, office, and workshop), number of work hours per day, and night shift. Night shift workers were working for varying times, starting as early as 19:30 and finishing as late as 08:30 the next morning on a fixed or rotational basis.

#### 2.2.3. Other Covariates

Socio-demographic characteristics and lifestyle factors were collected via self-administered questionnaires, clinical measurements were performed by trained staff using standard tools and protocols [21], and fasting blood samples were analyzed for plasma glucose and serum lipids.

(a) Socio-demographic characteristics were age, gender (male and female), marital status (single and married), education (up to pre-college, and college or above), ethnicity (Chinese, Malays and Indians), and monthly income (<S$4000 and ≥S$4000). For analysis purposes, due to small numbers, Malays and Indians were combined and classified as “non-Chinese”.

(b) Lifestyle habits included smoking (non/ex-smoker and current smoker), alcohol drinking (non-drinker, drinks less than once a month, and drinks once or more than once a month), intake of foods rich in vitamin D such as whole eggs and milk (servings per day), taking vitamin D supplements (cod liver oil, fish oil, and other vitamin D containing supplements) or calcium supplements regularly, and physical activity. The intake of vitamin D rich foods was assessed using a food frequency questionnaire (FFQ), adapted from the FFQ used in Singapore’s National Nutrition Survey [22]. For analysis purposes, due to small numbers, those drinking alcohol less than once a month, or at least once a month were combined and classified as “alcohol drinker”. Physical activity performed during leisure time on a typical day in a usual week was measured using the Global Physical Activity Questionnaire [23]. Frequency (days) and duration (minutes) of activities were multiplied and summed to obtain total minutes of leisure-time physical activity per week.

(c) Clinical measurements included height, weight, and blood pressure (BP). Height was measured using a stadiometer (Seca 217, Hamburg, Germany) to the nearest 0.1 cm, and weight was measured in light clothing using a digital scale (Seca 874, Hamburg, Germany) to the nearest 0.1 kg in accordance with the WHO STEPS protocol [21]. Body mass index (BMI) was calculated as weight in kilograms divided by height in meters squared (kg/m^2^). BP was measured over the right arm using the appropriate cuff size with an automatic digital BP monitor (Dinamap Pro100V2; Criticon, Norderstedt, Germany). Three readings were taken at two-minute intervals, and the average of all three readings was considered as the BP of participants.

(d) Blood samples were processed using the hexokinase method for plasma glucose and enzymatic methods for serum lipids on a COBAS 6000 analyzer, using kits supplied by Roche Diagnostics, Switzerland.

### 2.3. Statistical Analysis

The characteristics of participants are summarized using mean (SD) or median (inter-quartile range) for continuous variables and frequency and percentage for categorical variables. Bivariate and multivariate Poisson regression analyses with robust variance based on Huber’s sandwich estimator [24] were performed to examine the association between vitamin D deficiency and work-related factors. The results of Poisson regression are presented as prevalence ratios (PRs), 95% confidence intervals (CIs), and *p*-values. Potential confounders for multivariate modeling were chosen based on those factors that were previously identified in the literature, or variables with *p* < 0.20 or those that changed the effect size of exposures of interest (e.g., work-related factors) by >10% in bivariate analysis. Skewed variables were log-transformed for bivariate and multivariate analysis. The performance of the multivariate model was assessed using C-statistic for discrimination and Hosmer-Lemeshow goodness-of-fit test for calibration. A two-tailed *p*-value < 0.05 was considered for statistically significant results. All statistical analyses were conducted using Stata software version 15.0 (StataCorp, College Station, TX, USA).

### 2.4. Ethics Approval

The study was conducted in accordance with the Declaration of Helsinki and was approved by the Institutional Review Board of Nanyang Technological University Singapore, Singapore (IRB-2015-11-028). Written informed consent was obtained from all study participants prior to the commencement of data collection.

## 3. Results

Table 1 shows the characteristics of participants. The mean age was 42.5 years (SD: 11.0), and 76.5% were male. Slightly more than one-third (35.7%) were working in underground spaces. Workers were employed for a median of five years, working for an average of 8.6 h a day and were doing a median of one night shift a month.

The mean serum 25(OH)D level of participants was 59.6 nmol/L (SD: 21.8 nmol/L). The prevalence of vitamin D deficiency was 32.9% (95% CI: 26.6–39.6%). Table 2 shows the results of bivariate analysis of the association between vitamin D deficiency and covariates. Ethnicity, BMI, fasting plasma glucose, and workshop workers (versus control room workers) showed significant associations (all *p* < 0.05). 

Multivariate Poisson regression analysis showed that vitamin D deficiency was significantly associated with job type and night shift work after adjusting for socio-demographic characteristics, lifestyle habits, and clinical and biochemical characteristics (Table 3). The prevalence of vitamin D deficiency was 2.16 times (PR: 2.16, 95% CI: 1.12–4.16) and 2.25 times (PR: 2.25, 95% CI: 1.05–4.81) higher in office and workshop workers, respectively, compared to control room workers. Night shift work showed a significant PR of 1.31 (95% CI: 1.03–1.67) for vitamin D deficiency. Other work-related factors were not significantly associated with vitamin D deficiency.

## 4. Discussion

To our knowledge, this is the first study to examine the prevalence of vitamin D deficiency and its associated work-related factors among indoor workers in Singapore. On average, our study participants had the optimal level of serum 25(OH)D concentrations for bone health (≥50 nmol/L) but below the recommended level to achieve broader health benefits (≥75 nmol/L) [1]. The prevalence of vitamin D deficiency was 32.9%. Office and workshop workers (versus control room workers) and night shift workers were at a higher risk for vitamin D deficiency, after adjusting for socio-demographic characteristics, lifestyle habits, and clinical and biochemical characteristics.

The prevalence of vitamin D deficiency in our study (32.9%) was lower than the prevalence (42.1%) reported in the general population (mean age: 31.5 years) of Singapore [11]. This could be partly due to the lower proportion of females (22%) in our study compared to the general population study (48%). Females tend to have lower vitamin D levels than males, owing to the frequent use of sunscreen and higher body fat percentage [11]. There have been a few studies conducted in workplace settings on vitamin D status in Southeast Asia. Moy et al. examined 380 Malay employees aged 35 years and above in a public university in Malaysia. Nearly 70% of their study participants had vitamin D deficiency [14]. Yosephin et al. showed that 82% of garment factory female workers of reproductive age in Indonesia had serum 25(OH)D concentrations <75 nmol/L [25].

In our study, office and workshop workers were at a greater risk for vitamin D deficiency compared to control room workers. While offices have windows (closed), as UV-B is filtered by glass, indoor sunlight exposure does not produce vitamin D. There are no windows in control rooms, and lights are usually switched off to have a better visualization of the computer screens. Control room workers were doing more night shifts than office and workshop workers, which increases their risk for vitamin D deficiency. While the intake of vitamin D rich foods was similar across job types, control room workers were more likely to take vitamin D or calcium supplements, compared to office and workshop workers (Table S1). Control room workers have a rotation schedule, and at least two workers will be on duty at the same time. With this workplace practice, they are able to take regular and frequent breaks to go outdoors. Furthermore, conversation with control room employees revealed that they generally prefer to go outdoors for sunlight exposure due to their nature of work in dark environments. These factors probably offset the higher risk of vitamin D deficiency in control room workers due to night shifts [26,27] Night shift workers, due to their working hours and daytime sleeping, are generally exposed less to solar UV-B irradiation [28].

Previous studies have shown a positive correlation between work hours and vitamin D status [17,29]. Those who work for long hours indoors are likely to have low sunlight exposure, and thus are more prone to vitamin D deficiency. However, in our study, work hours was not significantly associated with vitamin D deficiency. This could be partly because of the small sample size with limited power to detect a significant difference. Self-reported nature of the work hours data may also have played a role.

Our study has some limitations. Firstly, since this was a cross-sectional analysis, a causal relationship between work-related factors and vitamin D deficiency could not be established. Secondly, the study’s sample size was small, resulting in wide confidence intervals, and some covariates possibly losing significance due to reduced statistical power. Therefore, our study findings should be interpreted with caution in view of this limitation. Nevertheless, our multivariate model had good discrimination (C-statistic: 0.81), and there was no evidence of poor fit (Hosmer–Lemeshow goodness-of-fit *p*-value: 1.00). Future studies with larger sample size are required to confirm our study findings. Finally, data on certain known confounders such as sunscreen use, time spent outdoors, intake of certain foods rich in vitamin D (e.g., oily fish, mushrooms), and renal, thyroid or parathyroid diseases as well as dietary practices (e.g., vegan diet) were not collected. Therefore, the possibility of residual confounding due to these variables cannot be excluded.

## 5. Conclusions

To conclude, the mean serum 25(OH)D concentration of our study participants was below the level considered optimal for general health, and one-third were in the deficient category. Office and workshop workers (versus control room workers) and night shift workers were at a higher risk for vitamin D deficiency. Workplace policies and wellness programs should incorporate regular screening programs for vitamin D in office, workshop and night shift workers. Workers should be encouraged to take breaks to go outdoors for sunlight exposure and to consume adequate amounts of vitamin D-rich foodsto maintain optimal vitamin D levels, although the role of supplements remains controversial.

## Figures and Tables

**Table 1:** Characteristics of the study participants.

Variables	*n* = 213
***Socio-demographic characteristics***	
Age (years)	42.5 ± 11.0
Gender	
Male	163 (76.5)
Female	50 (23.5)
Ethnicity ^a^	
Chinese	151 (70.9)
Non-Chinese	62 (29.1)
Marital status	
Single ^b^	72 (33.8)
Married	141 (66.2)
Education	
Up to pre-college	135 (63.4)
College or above	78 (36.6)
Monthly income	
<S$4000	141 (66.2)
≥S$4000	72 (33.8)
***Lifestyle habits***	
Smoking status	
Non/ex-smoker	176 (82.6)
Current smoker	37 (17.4)
Alcohol drinking	
Non-drinker	109 (51.2)
Drinker ^c^	104 (48.8)
Total minutes of leisure-time physical activity	90 (0–240)
Intake of vitamin D rich foods (servings/day) ^d^	0.7 (0.4–1.3)
Regular use of vitamin D or calcium supplements ^e^	
No	199 (93.4)
Yes	14 (6.6)
***Clinical and biochemical characteristics***	
Body mass index (kg/m^2^)	25.9 ± 5.5
Systolic blood pressure (mmHg)	122.9 ± 16.2
Diastolic blood pressure (mmHg)	73.1 ± 11.4
Fasting plasma glucose (mmol/L)	5.5 ± 1.4
Total cholesterol (mmol/L)	5.4 ± 1.1
***Work-related factors***	
Years employed in the current company	5.0 (2.7–10.8)
Work location	
Aboveground	137 (64.3)
Underground	76 (35.7)
Job type	
Control room worker	54 (25.3)
Office worker	109 (51.2)
Workshop worker	50 (23.5)
Work hours/day	8.6 ± 1.3
Night shifts per month	1 (1–1)

Data are mean ± standard deviation, median (inter-quartile range), or *n* (%). ^a^ Chinese includes a small number of mixed ethnicities (Filipino Chinese) and Filipinos. Non-Chinese includes Indians and Malays; ^b^ Includes never married, widowed, and divorced; ^c^ Includes those who drank alcohol less than once a month or at least once a month; ^d^ Includes milk and whole eggs; ^e^ Includes cod liver oil, fish oil, and other vitamin D containing supplements or calcium supplements.

**Table 2:** Association of vitamin D deficiency with covariates; results of bivariate analysis.

Variables	Prevalence of Vitamin D Deficiency	Unadjusted PR (95% CI)	*p*-Value
**Socio-demographic characteristics**			
Age (years)	-	1.00 (0.98–1.02)	0.89
Gender			
Male	32.5	1.00	
Female	34.0	1.05 (0.67–1.63)	0.84
Ethnicity ^a^			
Chinese	21.9	1.00	
Non-Chinese	59.7	2.73 (1.90–3.93)	<0.001
Marital status			
Single ^b^	34.7	1.00	
Married	31.9	0.92 (0.62–1.37)	0.68
Education			
Up to pre-college	37.0	1.00	
College or above	25.6	0.69 (0.45–1.07)	0.10
Monthly income			
<S$4000	37.6	1.00	
≥S$4000	23.6	0.63 (0.39–1.00)	0.05
***Lifestyle habits***			
Smoking status			
Non/ex-smoker	32.4	1.00	
Current smoker	35.1	1.08 (0.67–1.77)	0.74
Alcohol drinking			
Non-drinker	35.8	1.00	
Drinker ^c^	29.8	0.83 (0.56–1.23)	0.36
Total minutes of leisure-time physical activity	-	0.94 (0.87–1.00)	0.08
Intake of vitamin D rich foods (servings/day) ^d^	-	0.84 (0.52–1.33)	0.45
Regular use of vitamin D or calcium supplements ^e^			
No	34.7	1.00	
Yes	7.1	0.21 (0.03–1.38)	0.10
***Clinical and biochemical characteristics***			
Body mass index (kg/m^2^)	-	1.07 (1.04–1.10)	<0.001
Systolic blood pressure (mmHg)	-	1.01 (1.00–1.02)	0.05
Diastolic blood pressure (mmHg)	-	1.00 (0.99–1.02)	0.62
Fasting plasma glucose (mmol/L)	-	1.13 (1.07–1.18)	<0.001
Total cholesterol (mmol/L)	-	0.94 (0.78–1.14)	0.54
***Work-related factors***			
Years employed in the current company	-	0.93 (0.77–1.11)	0.41
Work location			
Aboveground	35.0	1.00	
Underground	29.0	0.83 (0.54–1.26)	0.37
Job type			
Control room worker	24.1	1.00	
Office worker	31.2	1.30 (0.75–2.25)	0.36
Workshop worker	46.0	1.91 (1.09–3.35)	0.024
Work hours/day	-	0.92 (0.79–1.08)	0.31
Night shifts per month	-	1.02 (0.83–1.25)	0.87

PR—prevalence ratio; CI—confidence interval. ^a^ Chinese includes a small number of mixed ethnicities (Filipino Chinese) and Filipinos. Non-Chinese includes Indians and Malays; ^b^ Includes never married, widowed, and divorced; ^c^ Includes those who drank alcohol less than once a month or at least once a month; ^d^ Includes milk and whole eggs; ^e^ Includes cod liver oil, fish oil, and other vitamin D containing supplements or calcium supplements.

**Table 3:** Work-related factors associated with vitamin D deficiency; results of multivariate analysis.

Work-Related Factors	Prevalence Ratio (95% CI)	*p*-Value
Years employed in the current company	1.02 (0.75–1.38)	0.92
Work location		
Aboveground	1.00	
Underground	0.89 (0.58–1.36)	0.59
Job type		
Control room worker	1.00	
Office worker	2.16 (1.12–4.16)	0.021
Workshop worker	2.25 (1.05–4.81)	0.037
Work hours/day	1.02 (0.86–1.22)	0.82
Night shifts per month	1.31 (1.03–1.67)	0.027

CI—confidence interval. Prevalence ratios were adjusted for age, gender, ethnicity, education, monthly income, leisure-time physical activity, intake of vitamin D rich foods, regular intake of vitamin D or calcium supplements, body mass index, systolic blood pressure, fasting plasma glucose, and total cholesterol. C-statistic: 0.81 (95% CI: 0.74–0.87) and Hosmer–Lemeshow goodness-of-fit: χ^2^: 132.2, *p* = 1.00.

## Supplementary Materials

The following are available online at https://www.mdpi.com/1660-4601/17/1/164/s1, Table S1: Characteristics of the study participants by job type.

## Abbreviations

UV-B.	ultraviolet B
25(OH)D	25-hydroxyvitamin D
BP	blood pressure
SD	standard deviation
PR	prevalence ratio
CI	confidence interval
