# Supplementary material for: Prevalence of Vitamin D Deficiency and Its Associated Work-Related Factors among Indoor Workers in a Multi-Ethnic Southeast Asian Country
Source: Int J Environ Res Public Health. 2019 Dec 25;17(1):164. doi: 10.3390/ijerph17010164 (PMC6981433; doi:10.3390/ijerph17010164)
Supplement: Supplementary file 1 [file ijerph-17-00164-s001.pdf]

Table S1. Characteristics of the study participants by job type.

	Office Workers	Control Room Workers	Workshop Workers
N	109	54	50
Socio-demographic characteristics			
Age (years)	41.9 ± 11.0	44.7 ± 11.4	41.4 ± 10.4
Gender			
Male	67 (61.5)	46 (85.2)	50 (100.0)
Female	42 (38.5)	8 (14.8)	0 (0.0)
Ethnicity [a]			
Chinese	89 (81.7)	42 (77.8)	20 (40.0)
Non-Chinese	20 (18.4)	12 (22.2)	30 (60.0)
Marital status			
Single [b]	37 (33.9)	21 (38.9)	14 (28.0)
Married	72 (66.1)	33 (61.1)	36 (72.0)
Education			
Up to pre-college	53 (48.6)	39 (72.2)	43 (86.0)
College and above	56 (51.4)	15 (27.8)	7 (14.0)
Monthly income			
<S$4000	62 (56.9)	30 (55.6)	49 (98.0)
≥S$4000	47 (43.1)	24 (44.4)	1 (2.0)
Lifestyle habits			
Smoking status			
Non/ex-smoker	100 (91.7)	43 (79.6)	33 (66.0)
Current smoker	9 (8.3)	11 (20.4)	17 (34.0)
Alcohol drinking			
Non-drinker	55 (50.5)	24 (44.4)	30 (60.0)
Drinker [c]	54 (49.5)	30 (55.6)	20 (40.0)
Total minutes of leisure-time physical activity	90 (0–240)	85 (0–150)	60 (0–330)
Intake of vitamin D rich foods (servings/day) [d]	0.7 (0.3–1.3)	0.7 (0.6–1.0)	0.9 (0.4–2.1)
Regular use of vitamin D or calcium supplements [e]			
No	102 (93.6)	48 (88.9)	49 (98.0)
Yes	7 (6.4)	6 (11.1)	1 (2.0)
Clinical and biochemical characteristics			
Body mass index (kg/m^2)	25.0 ± 5.1	26.3 ± 6.2	27.5 ± 5.3
Systolic blood pressure (mmHg)	119.3 ± 14.5	127.0 ± 17.1	126.1 ± 17.3
Diastolic blood pressure (mmHg)	70.5 ± 10.4	75.4 ± 12.2	76.2 ± 11.6
Fasting plasma glucose (mmol/l)	5.4 ± 1.5	5.6 ± 1.3	5.6 ± 1.3
Total cholesterol (mmol/l)	5.3 ± 1.1	5.5 ± 1.1	5.4 ± 0.9
Work-related factors			
Years employed in the current company	5.1 (3.0–9.3)	10.4 (3.2–30.1)	3.9 (1.7–5.3)
Work location			
Aboveground	74 (67.9)	34 (63.0)	29 (58.0)
Underground	35 (32.1)	20 (37.0)	21 (42.0)
Work hours/day	8.6 ± 0.9	9.3 ± 1.7	7.9 ± 1.2
Night shifts per month	1 (1–1)	8 (1–9)	1 (1–1)

Data are mean ± standard deviation, median (inter-quartile range) or n (%). [a] Chinese includes a small number of mixed ethnicities (Filipino Chinese), and Filipinos. Non-Chinese includes Indians and Malays. [b] Includes never married, widowed, and divorced. [c] Includes those who drank alcohol less than once a month or at least once a month. [d] Includes milk and whole eggs. [e] Includes cod liver oil, fish oil, and other vitamin D containing supplements or calcium supplements.
